# Motor vehicle crash reconstruction: Does it relate to the heterogeneity of whiplash recovery?

**DOI:** 10.1371/journal.pone.0225686

**Published:** 2019-12-04

**Authors:** James M. Elliott, Brad E. Heinrichs, David M. Walton, Todd B. Parrish, D. Mark Courtney, Andrew C. Smith, Jasmine Hunt, Mary J. Kwasny, Marie Wasielewski, Gunter P. Siegmund

**Affiliations:** 1 Faculty of Health Sciences, The University of Sydney & the Northern Sydney Local Health District, The Kolling Research Institute, St Leonards, NSW, Australia; 2 Department of Physical Therapy and Human Movement Sciences, Feinberg School of Medicine, Northwestern University, Chicago, IL, United States of America; 3 MEA Forensic Engineers & Scientists, Richmond, British Columbia, Canada; 4 School of Physical Therapy, Western University, London, Ontario, Canada; 5 Department of Radiology, Feinberg School of Medicine, Northwestern University, Chicago IL, United States of America; 6 Department of Emergency Medicine, Feinberg School of Medicine, Northwestern University, Chicago, IL, United States of America; 7 School of Physical Therapy, Regis University, Denver, CO, United States of America; 8 Department of Preventive Medicine, Feinberg School of Medicine, Northwestern University, Chicago, IL, United States of America; 9 School of Kinesiology, University of British Columbia, Vancouver, British Columbia, Canada; Monash University, AUSTRALIA

## Abstract

Whiplash injury is a common consequence of motor vehicle crashes (MVC), yet it is also one of the most poorly understood. While more than 50% of those injured should expect to rapidly recover, others are not as fortunate with approximately 25% of those exposed to and injured in an MVC transitioning from acute to chronic pain and disability. The purpose of this prospective study was to determine if the severity and direction of collisions involving participants enrolled in a longitudinal study of recovery from whiplash are able to differentiate between different recovery groups based on the neck disability index (NDI) percentage scores at 3-months, and if these crash specific parameters are associated with known risk factors for recovery. Here, we examined objective collision data, repair invoices, and characteristics of the crash for 37 acutely injured participants consented and enrolled at their emergency department visit and further assessed at three time points; < 1 week, 2-weeks, and 3-months post MVC. Collision data were used to reconstruct and estimate the severity of the crash and determine if they aligned with the heterogeneity of whiplash injury recovery. Wilcoxon rank sum tests were used to determine if % scores on the Neck Disability Index (NDI) at 3-months post MVC were associated with the following variables: sex, head turned at time of impact, seatbelt use, whether or not airbags deployed, if the vehicle was struck while stopped or while turning, or the principle direction of force (PDOF). Spearman’s correlation coefficients were used to determine if NDI at 3-months post MVC was associated with age, Body Mass Index, pain-related disability at baseline, signs of post-traumatic distress, intrusion/hyperarousal, negative affect, pain intensity, estimated speed change from the impact, and damage estimates (in US$). There was a significant positive association between self-reported neck disability at 3-months post MVC, post-traumatic distress, negative affect and uncontrolled pain. There was no direct effect of participant characteristics, arousal, intrusion/hyperarousal sub-score, damage, PDOF, speed change, or other crash characteristics. Established crash parameters were not associated with the heterogeneity of whiplash injury recovery in a small sample of injured participants.

## Introduction

Neck pain and related symptoms arising from non-catastrophic injuries following a motor vehicle collision (colloquially known as whiplash injury) can significantly influence quality of life for some, but not all, vehicle occupants. While the collision-related mechanics of the head and neck gave rise to the term whiplash,[1, 2] the signs and symptoms developing in victims of these crashes are often complex and clinically known as whiplash-associated disorders (WAD).[3]

Evidence suggests up to 50% of acutely injured people will fail to fully recover,[4] with approximately 25% demonstrating a markedly complex clinical picture including higher levels of neck-related interference,[5, 6] higher levels of reported pain intensity,[6] muscle composition changes,[7–14] sensory and motor disturbances,[15–20] and muscle weakness.[21] A number of other psychosocial factors (e.g., early pain poor coping, little expectation of recovery, influence of compensation[22, 23] higher anxiety and depression)[24] have also shown to influence recovery rates, suggesting a role for both medical and non-injury related factors.[25] However, despite the presence and recognition of these factors, current best multimodal management options (e.g. physical therapies, pharmacological agents, and psychological regimens) have not substantially influenced the recovery rates for all of those exposed and injured.[26–28]

Those with chronic WAD, and those treating patients with this condition, would benefit greatly if quantitative biomarkers related to persistent signs and symptoms were available, but the identification of relevant biologics or structural pathologies of discs, ligaments, vertebral arteries, muscles, and facet joints have remained elusive.[29] To date, the most complete patho-mechano-physiological explanation for chronic whiplash pain has been assembled for the facet capsular ligament. Sub-catastrophic ligament failures have been generated in cadaveric tissues following whiplash-like loading;[30–32] nociceptor activation occurs when the facet capsular ligament is stretched in an in-vivo caprine model;[33] chronic pain states have been shown by straining this ligament in an in-vivo rodent model;[30] and successful diagnostic blocks and ablation treatments targeting these tissues have shown to improve pain and negative affect in a sub-group of human participants.[34–36] The picture for other organic lesions, however, remains far less complete.[29]

Attempts to study the underlying causal factors of the incident event preceding WAD often involve crash reports from the occupant(s) of the struck and striking vehicles, police reports, and/or witness accounts. This information typically includes *direction of impact*, *presence and type of head restraint*, *head position/posture at time of impact*, *and preparedness for the impact*. As self-reported collision data and witness reports are subjective, and vulnerable to recall errors, more quantitative collision data could shed light on the link between the parameters of the crash, occupant kinematics, potential injury mechanisms, and findings from imaging and clinical tests. For example, collision data such as photos, repair estimates, event data recorders (EDRs), and repair invoices of both involved vehicles can be used by a crash reconstructionist to estimate collision severity.

While various measures of collision severity have been used to generate estimates of occupant injury risk,[37–39] these analyses have not been combined with acknowledged biopsychosocial risk factors of recovery from whiplash. The primary purpose of our study was to determine if the severity and direction of collisions involving participants enrolled in a longitudinal study of recovery from whiplash are able to differentiate participants based on the neck disability index (NDI) percentage scores at 3-months and if these crash specific parameters are associated with known risk factors for recovery.

## Materials and methods

Participants presenting to a large academic emergency medicine department with level 1 trauma designation in Chicago, IL, USA enrolled in a parent longitudinal cohort study investigating recovery from whiplash injury (ClinicalTrials.gov Identifier: NCT02157038). Participants were eligible provided they reported neck pain resulting from a motor vehicle collision and were willing to participate in the crash reconstruction portion of the parent longitudinal study involving 97 acutely injured participants. Thirty-seven of 97 participants were eligible and consented to participate. Later, each participant also provided vehicle repair invoices and further demographics of the crash event. Following their emergency department visit, all participants were further assessed at three time points: < 1 week, with follow-up assessments at 2-weeks post MVC, and 3-months post MVC.

The Institutional Review Board of Northwestern University, Feinberg School of Medicine granted approval (STU00090769) and all participants provided informed written consent. Exclusion criteria were younger than 18 or older than 65 years of age, one or more previous motor vehicle collisions in their lifetime, treatment for neck pain disorders in the past ten years, any nervous system disorders (e.g. stroke, Parkinson’s), metabolic system disorders (e.g. diabetes), or those who, by standard Emergency Department protocols, were deemed to be at risk for multi-system trauma.

One research assistant (MW) administered questionnaires to all subjects at each assessment. One of the authors (BEH), a professional engineer, blind to the clinical status of the vehicle occupants, reconstructed all of the crashes. The reconstruction process consisted of reviewing all vehicle-related documentation and estimating the speed change (in km/h) and impact direction sustained by a subject’s vehicle during their crash. Vehicle speed change was calculated as the magnitude of the vector difference between a vehicle post-impact and pre-impact velocity. Impact direction was the angle in the horizontal plane of this same vector difference. Speed change is the standard measure of a crash severity from the perspective of an occupant inside the vehicle and, when combined with impact direction, determines the magnitude and direction of occupant motion within the vehicle immediately after the crash. Standard collision reconstruction methods were used, which consisted primarily of comparing the amount of vehicle damage to crash tests of known severity staged by the National Highway Traffic Safety Administration (NHTSA), Insurance Institute for Highway Safety (IIHS), Crash Test Service (CTS), Accident Reconstruction Network–Collision Safety Institute (ARC-CSI), and MEA Forensic. For eight of the reconstructions, PC-Crash (11.1. Dr. Steffan Datentechnik, Linz, Austria) was also used to assess vehicle motion and to bound speed change in crashes with more than two vehicles. For each crash, a minimum and maximum speed change was estimated using the available data, and then a single value that represented the reconstructionist’s best estimate within this range was also generated. The principal direction of the impact force (PDOF) applied to a subject’s vehicle was categorized as either from the rear (impact angles <-135° or >135°; with 0° being a straight frontal impact and ±180° being a straight rear-end impact) or from any direction other-than-rear (-135°<PDOF<135°).

### Subjective (self-reported) clinical outcomes

#### Primary Outcome: Self-reported neck-related disability at 3-months post MVC

Self-reported neck-related disability was measured using the NDI administered at baseline (< 1-week), 2-weeks, and 3-months post MVC. The NDI has been used extensively across populations of patients with traumatic and non-traumatic neck pain.[9, 11, 19, 26, 27] As different thresholds for low, moderate, and high disability have been provided by prior authors, we have used this as a continuous measure of disability.

**The Numeric Pain Rating Scale (NPRS)** is a self-report unidimensional measure of pain intensity in which the respondent selects a whole number (0–10 integers) that best reflects the intensity of their pain.[6, 40]

### Psychological Distress

**The Traumatic Injuries Distress Scale (TIDS)** is a 12-item self-report tool with 3 subscales (negative affect, uncontrolled pain, intrusion/hyperarousal). It has demonstrated adequate internal and longitudinal properties and offers estimates of both magnitude and nature of risk of chronic problems following musculoskeletal injury.[41]

### Statistical analyses

Baseline demographics and disability data were compared between the 37 participants who agreed to be in this sub-component of the parent study, and those who did not wish to participate (n of 60) using chi-squared (sex) and t-tests (age, BMI, NDI, compensation claim lodgement and whether the participant engaged legal services at 3-months post-MVC). Characteristics of the crash are presented using counts and percentages and means and standard deviations for all clinical outcomes assessed at the three time points are compared using a repeated measures ANOVA. When comparing associations between these measures, as NDI is skewed, non-parametric methods were used to assess if baseline characteristics or any of the crash reconstruction variables were associated with 3-month disability. Specifically, Wilcoxon rank sum tests were used to determine if disability at 3-months post MVC was associated with sex, whether the person’s head was turned at time of impact, seatbelt use, whether or not airbags deployed, if the vehicle was struck while stopped or while turning, the PDOF, or litigation status. Spearman’s correlation coefficient was estimated to determine if disability at 3-months post MVC was associated with age, BMI, PDS-Arousal, pain-related disability at baseline, TIDS total and sub-scores, estimated speed change (kilometers per hour–km/h) from the impact, and estimated damage costs (in US$). Given the smaller sample size, and the fact that this is an exploratory sub-study of a larger cohort, all tests were conducted at a type I error rate of 5%, and all tests were performed in SAS v9.4 (SAS Institute, Cary, NC).

## Results and discussion

A total of 37/97 (38%) participants provided consent for a field engineer to procure multiple photographs of their damaged vehicle before it was repaired. The demographic characteristics of the participants in the sub-study was similar to those from the entire study (Table 1). Crash characteristics are presented in Table 2 and Table 3 details clinical outcomes over time for all participants. There was no direct effect of participant characteristics (age, BMI, sex), PDS Arousal, TIDS intrusion/hyperarousal sub-score, damage, PDOF, speed change, or other crash characteristics (Tables 4 and 5) on self-reported neck disability. The median NDI % score for those engaging a lawyer was significantly higher than for those not engaging a lawyer (Table 4). There was a significant positive correlation between self-reported neck disability and pain-related disability at baseline, TIDS total score, TIDS negative affect, and TIDS uncontrolled pain sub-scores (Table 5).

**Table 1:** Group descriptives—data for age, BMI, NDI, compensation claim lodgement status and engaging legal services (%) at 3-months for those in, and those not in, the sub-study investigating crash parameters (displayed as mean (SD)).

Baseline Characteristic	In sub-study	Not in sub-study
N	37	60
Sex (Female)	28 (76%)	44 (73%)
Age	37 (11)	33 (11)
BMI	24.5 (3.8)	26.6 (7.0)
NDI	38 (19)	34 (14)
Claim Lodgement (Yes %)	5.4	21.67
Engaged Legal services (Yes %)	43.2	33.3

BMI–Body Mass Index

NDI–Neck Disability Index

**Table 2:** Characteristics of the crashes are presented using counts (percentages), or mean ± standard (range).

Variable	n(%)
*PDOF (rear >135, <-135)	20 (54%)
Head turned	18 (49%)
Seatbelt on	25 (68%)
Airbags deployed	12 (32%)
Struck while stopped	14 (38%)
Struck while braking	20 (54%)
Low estimate speed (km/h)	10.8 ± 5.6 (0–25)
High estimate speed (km/h)	25.4 ± 11.1 (10–56)
Best estimate speed (km/h)	17.2 ± 7.1 (6–35)

*PDOF–Principle Direction of Force

**Table 3:** clinical outcomes assessed at the three time points for all 37 participants. Data displayed as means (standard deviations).

Variable	<1 week	2 weeks post	3 months post	p-value
NDI(%)	37.8 (19.2)	30.3 (18.5)	18.4 (17.1)	<0.001
PDS (arousal)	4.3 (4.0)	4.6 (4.0)	3.1 (3.3)	0.034
Pain Intensity (NPRS)	5.2 (2.4)	4.4 (2.8)	3.0 (2.8)	<0.001
TIDS-total	9.9 (6.5)	8.3 (6.9)	6.5 (6.1)	0.008
TIDS-negative affect	4.2 (3.5)	3.8 (3.5)	3.4 (3.3)	0.431
TIDS-uncontrolled pain	3.9 (2.8)	3.2 (2.8)	1.9 (2.5)	<0.001
TIDS-intrusion/hyperarousal	1.8 (1.4)	1.4 (1.4)	1.2 (1.3)	0.019

PDS–Post-Traumatic Diagnostic Scale

TIDS–Traumatic Injury Distress Scale

**Table 4:** Categorical associations with NDI.

Characteristic	Median NDI (25th, 75th) [min, max]	p-value
Male Female	8 (4, 8) [0, 46] 17 (6, 17) [0, 76]	0.33
PDOF (rear >135, <-135) Other PDOF	17 (5, 17) [2, 76] 10 (6, 10) [0, 56]	0.58
Head turned Head not turned./unknown	17 (6, 17) [0, 56] 10 (4, 10) [2, 76]	0.96
Seatbelt on Seatbelt not on/unknown	12 (6, 12) [0, 76] 13 (3, 13) [0, 46]	0.45
Airbags deployed Airbags not deployed/unknown	11 (5, 11) [0, 76] 16 (6, 16) [0, 46]	0.83
Struck while stopped Struck while moving	8 (4, 8) [0, 32] 16 (6, 16) [0, 76]	0.22
Struck while braking Not braking	14 (4, 140 [0, 46] 12 (8, 12) [0, 76]	0.60
Engaged lawyer Did not engage lawyer	20 (16, 32) [6, 76] 6 (4,10) [0, 46]	0.001

**Table 5:** Spearman’s rank correlations with NDI.

Characteristic	Spearman’s correlation r_s_ (95% CI)	p-value
Age	0.11 (-0.22, 0.42)	0.50
BMI	-0.09 (-0.41, 0.24)	0.57
PDS Arousal	0.24 (-0.09, 0.53)	0.14
Pain-related disability (baseline)	0.48 (0.19, 0.70)	0.002
TIDS Total	0.46 (0.16, 0.68)	0.004
TIDS Negative affect	0.36 (0.04, 061)	0.03
TIDS uncontrolled pain	0.55 (0.27, 0.74)	<0.001
TIDS Intrusion/Hyperarousal	0.17 (-0.16, 0.47)	0.31
Low estimate Speed	-0.25 (-0.53, 0.08)	0.13
High estimate Speed	-0.26 (-0.54, 0.07)	0.12
Best estimate Speed	-0.24 (-0.53, 0.09)	0.14
Damage	-0.10 (-0.48, 0.30)	0.61

We captured and reported on established crash parameters and a PDOF estimate to determine if they directly aligned with the heterogeneity of WAD recovery in a sample of 37 acutely injured participants, but an association was not detected. This finding is perhaps not surprising given the wide range of crash severities that have been reported to cause and not cause whiplash injury. For example, Krafft et al.[37] reported that crashes with speed changes as low as 8 km/h resulted in whiplash symptoms lasting longer than 6 months in some individuals, whereas crashes with speed changes as high as 17 km/h resulted in no whiplash symptoms in other individuals. We sought to expand on their injury data, which consisted only of symptom duration (<1 month, 1–6 months, and > 6 month) and symptom severity (WAD 0 through WAD 3), by including established research- and clinical-based measures such as psychological distress (PDS (arousal) & TIDS scores), and demographics (age, sex, BMI), respectively. Nevertheless, we failed to detect significant correlations between crash parameters and self-reported neck disability levels despite the more detailed information. A significant difference in neck-related disability was however noted in that participants engaging a lawyer had significantly higher NDI scores when compared to those who did not engage a lawyer. While it is possible that some people involved in litigation could plausibly exaggerate the duration and intensity of their symptoms, there is no clear or consistent evidence to support the idea that compensation, litigation, and their related processes leads to worse health outcomes. [23]

The current work does support a prevailing view that there is little correlation between the severity of low-speed crashes and the severity/duration of the patient’s injury or WAD-related symptoms. The current study does not disprove the existence of a possible dose-response relationship between crash severity and whiplash symptoms, though is perhaps better conceptualized as further support that the spectrum of WAD symptoms is a function of more than crash parameters alone. In the context of this study, it is arguably unrealistic to expect simple, unadjusted, linear bivariate relationships between parameters like the speed of the crash and the magnitude of functional interference reported by patients. From the clinical side, there is no objective gold-standard diagnostic option for whiplash injury and no objective means of grading its severity. From the exposure perspective, there are a number of difficult-to-quantify factors related to a patient’s initial posture and preparedness alone that could alter the forces developing in and potentially injuring soft-tissues about the spine. From a tolerance perspective, we know that the range of tolerance levels for soft-tissue injury is wide. Indeed, tolerance levels for micro-failures of the facet capsule (a known source of chronic pain in patients with whiplash) varies by more than a factor of four in females.[42] And finally, from a coping and healing—*or return to pre-injury status*—perspective, there are many psychosocial factors that affect recovery trajectories.[4, 22, 24, 25] When viewed from these broad perspectives, it may be overly optimistic to expect the existence of a global dose-response relationship between low-resolution crash parameters and patient outcomes. Accordingly, caution should be exercised when attempting to establish cause-and-effect with low-resolution crash reconstruction techniques in isolation and without consideration of other known bio-psychosocial risk factors on a patient-by-patient basis.

This study does, however, support the widely-reported multifactorial picture of whiplash recovery where both medical and non-injury related factors drive the clinical course,[25] and pre-existing health may also feature.[43] Here, we demonstrated temporal changes in a commonly used region-specific disability scale (NDI), and ongoing signs of arousal, traumatic distress, and uncontrolled pain in a small population of those with varying levels of whiplash-related disability. The results do not suggest the biomechanics of a motor vehicle collision are unrelated to injury risk, but they question the ability of low-resolution crash reconstruction methods commonly used in real-world clinical practice and medical-legal cases to predict likelihood of recovery.

The current study is limited by a reliance on patient self-reported recollection of the crash event (e.g. head turned at impact, seatbelt on, position of head-restraint at time of impact, etc). The absence of a relationship between the patient/clinical variables and the collision reconstruction variables may be due to the wide range of some of the severity estimates reported in this paper. This wide range resulted from the discrete nature of some collision-related damage wherein some vehicles or components do not show damage until a certain speed change is reached. In the absence of such damage, it is often not possible to determine how far below damage threshold the actual collision severity was. Most modern cars have EDRs installed as standard equipment and relying on these devices could improve the estimates of collision severity, collision direction, and seatbelt use. Many EDRs in the current North American fleet, however, only capture the collision severity for frontal crashes, and therefore the striking vehicle’s EDR data are needed to estimate the struck vehicle’s speed change in a rear-end crash. In this study, 40/78 of the involved vehicles had readable EDRs but we relied on traditional reconstruction techniques and patient recall because the EDR data was not made available. While these EDRs often need to be corrected for systematic errors,[44] future research should consider downloading these data to improve crash severity estimates and avoid the challenges associated with low-resolution reconstruction techniques.

## Conclusions

This preliminary work does not eliminate the possibility that crash-related factors influence the risk and subsequent recovery from whiplash injury. It does, however, increase confidence that crash severity estimates based on low-resolution crash reconstruction techniques in isolation do not uniquely inform rates of whiplash injury risk and recovery. We remain encouraged that further work using higher resolution data from EDRs in combination with acknowledged risk-factors may enrich our understanding of the bio-psycho-social factors driving the clinical course of and recovery from whiplash injury.

## Supporting information

S1 Data This is the Crash_outcomes_Final dataset. All data underlying the study are available within the manuscript and its Supporting Information files.
(XLS)
